# Anal and oral human papillomavirus (HPV) infection in HIV-infected subjects in northern Italy: a longitudinal cohort study among men who have sex with men

**DOI:** 10.1186/1471-2334-11-150

**Published:** 2011-05-25

**Authors:** Saverio G Parisi, Mario Cruciani, Renzo Scaggiante, Caterina Boldrin, Samantha Andreis, Federico Dal Bello, Silvana Pagni, Andrea Barelli, Andrea Sattin, Carlo Mengoli, Giorgio Palù

**Affiliations:** 1 Department of Histology, Microbiology and Medical Biotechnology, Padua University, Via Gabelli 63, 35100 Padova, Italy; 2 Center of Preventive Medicine and HIV Outpatient Clinic, ULSS 20, via Germania 20, 37135 Verona; 3 Infectious Diseases Unit, Padova Hospital, via Ospedale 1, 35100 Padova; 4 Infectious Diseases Unit, Venezia Mestre Hospital, via Paccagnella 11, 30100 Venezia Mestre

## Abstract

**Background:**

A study including 166 subjects was performed to investigate the frequency and persistence over a 6-month interval of concurrent oral and anal Human Papillomavirus (HPV) infections in Human Immunodeficiency Virus (HIV)-infected men who have sex with men (MSM).

**Methods:**

Patients with no previously documented HPV-related anogenital lesion/disease were recruited to participate in a longitudinal study. Polymerase chain reaction (PCR) was performed to detect HPV from oral and anal swabs and to detect Human Herpes Virus 8 (HHV-8) DNA in saliva on 2 separate specimen series, one collected at baseline and the other collected 6 months later. A multivariate logistic analysis was performed using anal HPV infection as the dependent variable versus a set of covariates: age, HIV plasma viral load, CD4+ count, hepatitis B virus (HBV) serology, hepatitis C virus (HCV) serology, syphilis serology and HHV-8 viral shedding. A stepwise elimination of covariates with a p-value > 0.1 was performed.

**Results:**

The overall prevalence of HPV did not vary significantly between the baseline and the follow-up, either in the oral (20.1 and 21.3%, respectively) or the anal specimens (88.6 and 86.3%). The prevalence of high-risk (HR) genotypes among the HPV-positive specimens was similar in the oral and anal infections (mean values 24.3% and 20.9%). Among 68 patients with either a HR, low-risk (LR) or undetermined genotype at baseline, 75% had persistent HPV and the persistence rates were 71.4% in HR infections and 76.7% in LR infections. There was a lack of genotype concordance between oral and anal HPV samples. The prevalence of HR HPV in anus appeared to be higher in the younger patients, peaking (> 25%) in the 43-50 years age group. A decrease of the high level of anal prevalence of all genotypes of HPV in the patients > 50 years was evident. HHV-8 oral shedding was positively related to HPV anal infection (p = 0.0046). A significant correlation was found between the persistence of HHV-8 shedding and HIV viral load by logistic bivariate analysis (Odds Ratio of HHV-8 persistence for 1-log increase of HIV viral load = 1.725 ± 0.397, p = 0.018).

**Conclusions:**

A high prevalence of HPV infection was found in our cohort of HIV-infected MSM, with a negative correlation between anal HPV infection and CD4 cell count.

## Background

Human papillomavirus infections are among the most common sexually transmitted infections worldwide, representing a significant health problem due to their high prevalence and transmissibility. HPV is a leading cause of anogenital malignancies. The incidence of anal cancer is particularly high among women with a history of cervical dysplasia and cervical cancer, HIV-positive individuals, Men who have sex with Men (MSM) and transplant recipients [1]. Anal cancer is increasingly recognised as an immunodeficiency-related cancer, and there is strong evidence that the incidence of anal cancer is stable or increasing in homosexual men and people with HIV infection, even in the highly active antiretroviral therapy (HAART) era [2-6].

Several studies have found high rates of anal HPV infections in HIV-infected patients [6-9]. Prevalence estimates vary according to the diagnostic methods and populations examined. MSM living with HIV are particularly susceptible to HPV infection, often involving multiple strains of HPV.

Recent molecular and epidemiological studies have demonstrated a role for HPV in the aetiology of oropharyngeal cancers [10,11]. High-risk (HR) HPV, predominantly type 16, has been consistently detected in a distinct subset of these cancers [10]. Despite these important observations, little is known about the epidemiology of oral HPV infection. Initial studies indicate that oral HPV infection, analogous to cervical infection, is associated with sexual behaviour and immunosuppression [12,13]. However, it is unclear to what extent data on cervical HPV infection can be extrapolated to oral HPV infection. Likewise, data on the spread of HPV infection to the various body parts implicated in sexual practices are limited.

HPV infection is associated with oral squamous cell carcinoma (OSCC). Although the incidence of HPV-unrelated OSCC associated with tobacco and alcohol use has declined over recent decades, OSCC related to HPV has significantly increased, particularly among white men and younger individuals [14]. This may be a result of changes in sexual behaviours. Given the incidence ratio of 2.32 for OSCC in HIV-infected persons, particular attention should be paid to this oral cancer in the setting of HIV infection, especially in HIV-positive MSM [3].

The few available studies analysing concurrent or sequential oral and anogenital HPV infections have used the similar study populations: high-risk sex workers, women with a history of cervical HPV infection, and HIV-positive women [15-18]. Several aspects of the relationship of oral HPV infection to anal HPV infection remain poorly described. These include differences in HPV prevalence and HPV type distribution by anatomic site, the prevalence of concomitant oral and anal infections, and whether concomitant infections are type concordant. Oral sex is a common practice among MSM and may lead to the transmission of various STDs [19-21].

This study was designed to investigate the frequency and persistence over a six-month interval of concurrent oral and anal HPV infection in a cohort of HIV-infected MSM and to investigate correlations between these infections and virological and immunological status related to HIV and other infections, including HHV-8, HBV, HCV, and syphilis.

## Methods

From December 2007 to June 2009, HIV-positive MSM attending the Infectious Disease Unit of the Padua University Hospital as outpatients were recruited to participate in a longitudinal study of HPV infection. Eligibility criteria included being > 18 years of age, able to provide informed consent and having no history of or current symptomatic HPV-related anogenital lesions/diseases. Each subject underwent clinical evaluation and sample collection on two different occasions separated by 6 months. HPV was assayed from oral and anal swabs.

All virologic and serologic tests were performed as a part of the diagnostic procedures performed periodically on these HIV patients. All subjects enrolled gave their informed consent to all procedures and to the use of their data for scientific evaluation and publication in a blinded form. The local government in the form of the Veneto Regional Health Authority approved this study and provided funds. This study was conducted in accordance with the Helsinki Declaration and with local legislation.

At each visit, a PBS-moistened anal Dacron swab was inserted 3 cm into the anal canal and then used to scrape along the anal walls by rotating three times clockwise and three times anticlockwise. Another swab was used for oral sampling; oral samples were obtained by vigorous scraping of the walls of the oropharyngeal cavity by rotating two times clockwise and two times anticlockwise. The swab samples were then agitated in 1.5 mL of 1 × PBS (Phosphate-Buffered Saline). Each sample was kept at 4°C and processed within four hours. After centrifugation at 13,000 *g *for 15 min at 22°C, the supernatant was discarded, the cell pellet was dried and resuspended in 300 ul of 20 mmol/L Tris buffer (pH 8.3) and then frozen at -80°C. Total DNA was extracted using the QIAamp DNA Mini Kit (Qiagen, GmbH, Hilden, Germany), and DNA extracted from the samples was first tested with primers PC04 and GH20, targeting a 268 bp fragment of beta-globin. Samples negative for beta-globin were not considered for further analysis. DNA was purified using ExoSAP-IT (USB Corporation, Cleveland, Ohio), and the presence of HPV DNA was investigated by PCR using MY09/MY11 primers and, if HPV DNA was not detected, GP5+/GP6+ primers [22-24]. Nested PCR was not used. To identify the HPV genotype, amplicons that were detected by agarose gel electrophoresis were subsequently submitted to sequencing by using the BigDye1 Terminator v3.1 Cycle Sequencing Kit on a 3100 Genetic Analyser (Applied Biosystems, Foster City, CA). After alignment using the SeqScape v2.5 software (Applied Biosystems), sequences were analysed in GenBank by using the NCBI BLAST tool. The risk level of each genotype was classified according to oncogenic properties in the cervix [25].

Thus, genotypes 10, 32, 34, 38, 43, 85, 86, 90, and 97 were considered of undefined oncogenic risk, 6, 11, 26, 40, 42, 53, 54, 55, 61, 62, 64, 66, 67, 69, 70, 71, 72, 73, 81 82, 83, 84, IS39 and CP6108 of low risk (LR), 16, 18, 31, 33, 35, 39, 45, 51, 52, 56, 58, 59, 68 and 73 of high risk.

HHV-8 DNA detection by PCR was also performed on the saliva from all patients. Briefly, DNA extraction and purification from the saliva samples (500 ul, processed as plasma samples) was performed using the QIAmp Blood kit (Qiagen, Inc., Chatsworth, CA). The detection of HHV-8 DNA in samples was performed using the real-time polymerase chain reaction (RT-PCR) using TaqMan probes (Applied Biosystems, Foster City, CA) of open reading frame 26 (ORF26) as previously described [26]. The nucleotide sequence targeted by the primers and probes is highly conserved among the three major subgroups of HHV-8 [27]. During all DNA extractions and purifications, precautions were taken to reduce the risk of false-positive results.

HIV plasma viral load (copies/ml) was evaluated using the Roche Cobas AmpliPrep-Cobas TaqMan HIV-1 assay, version 1 (F. Hoffmann-La Roche, Diagnostics Division, Basel, Switzerland).

### Data collection and statistical analysis

Data collected at study entry included age in years, whether the patient was receiving antiretroviral treatment, CD4 lymphocyte count (cells/μl), serology for *T. pallidum *infection, and HBV and HCV serology.

The PCR assays for HPV in oral and anal swabs and for HHV-8 in saliva were performed on 2 separate specimens, one collected at baseline and a second collected after a 6-month interval. The age, HIV viral load in plasma and CD4 count were considered as quantitative (continuous) variables, while the remainder of the variables were binary. The detection of HPV genomes was binary and positives were supplemented by genotype identification. The serology for syphilis included a non-treponemal test (Venereal Disease Research Laboratory (VDRL)), a treponemal test (Treponema hemagglutination test (TPHA)), and an ELISA screening test.

A multivariate logistic analysis was performed using the presence of anal HPV infection as a dependent variable versus a set of covariates including: age, HIV viral load, CD4 cell count, HBV serology, HCV serology, syphilis serology and HHV-8 viral shedding. The study participants' ages was broken into 4 groups (< 35, 35-42, 43-50, and > 50 years). A stepwise elimination of covariates with a p-value > 0.1 was adopted.

## Results

**Figure 1:**
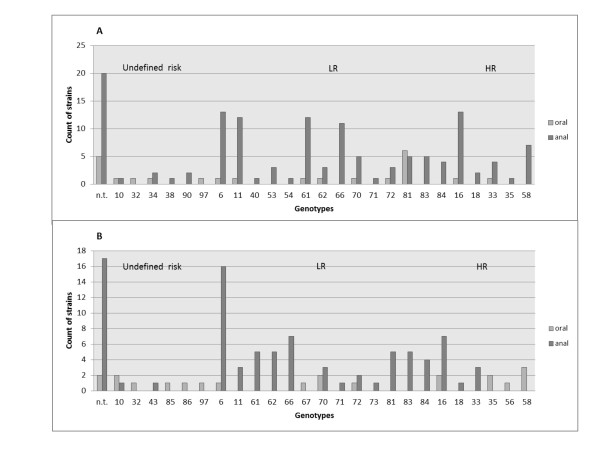
**HPV genotype distribution of all oral and anal infections detected among paired oral and anal swabs**. A: HPV genotype distribution at baseline; B: HPV genotype distribution in subsequent specimens (6 months after study entry). n.t.: non-typeable HPV strains. The genotype is indicated on the abscissa. Genotypes 10, 32, 34, 38, 90, (anal swabs) and 10, 97 (oral swabs) are of undefined oncogenic risk; genotypes 6, 11, 40, 53, 54, 61, 62, 66, 70, 71, 72, 81, 83, 84 (anal swabs) and 6, 66, 81 (oral swabs) are low-risk; genotypes 16, 18, 33, 35, 58 (anal swabs) and 16, 18, 33, 35, 56, 58, 73 (oral swabs) are high-risk.

A total of 166 men were enrolled in this study. The characteristics of the study population are listed in Table 1. Paired concurrent anal and oral swabs were collected from all 166 of the patients at baseline. There were 49 withdrawals from the study, meaning that specimens at the 6 month follow-up were available from only 117 patients. Moreover, 41 oral swabs (thirty-two at baseline: 19%, nine at follow-up: 7.7%) were not able to be evaluated by PCR due to inadequate sampling revealed by the inability to detect beta-globin in the DNA sample. All anal swabs were adequate for sampling. Therefore, 283 anal swabs and 242 oral swabs were available for analysis. The HPV detection in oral and anal swabs is indicated in Table 2. The overall prevalence of HPV did not vary significantly between the baseline and follow-up time points and was 20.1 vs. 21.3% in oral specimens ((p-value = 0.8266) and 88.6 vs. 86.3% in anal specimens (p-value = 0.5748). The prevalence of HR genotypes among HPV-positive specimens was similar in oral and anal infections (mean values 24.3% and 20.9%, p-value = 0.6417). Among the 68 patients with HR, LR, or undetermined genotypes at the baseline in their anus who provided two consecutive anal samples, 75% (51/68) had persistent HPV six months after enrolment. Persistence rates of 71.4% (10/14) in patients with high-risk infections and 76.7% (33/43) in patients with low-risk infections were found. Rates of persistence of HPV according to the level of risk associated with the HPV genotype are shown in Table 3. The HPV genotype distribution in oral and anal swabs is depicted in Figure 1. There was no genotype concordance between oral and anal HPV types, with the exception of non-typeable strains in 3 paired oral and anal swabs (in a single patient at baseline and in two patients at follow-up). Genotype identification was not performed when multiple co-infecting HPV strains were obtained (Table 2).

**Table 1:** Baseline characteristics of the study population

Age	
mean ± SD	43.8 (± 12.31)
median (range)	42 (21-80)
CD4 count	
mean (± SD)	563 (± 262)
median (range)	515 (0-1390)

HIV viral copies/ml	
mean ± SD	68.356 (± 250.712)
median (range)	372 (< 20-2,139.627)
viral load < 20 copies/ml: no. pts. (%)	38 (22.9)

Antiretroviral treatment: no. pts. (%)	
Yes	87 (63)
No	51 (37)
No data	28 (16.8%)

HHV-8 DNA shedding in saliva: no. pts. (%)	
Pos./neg.	36/105 (25.5/74.5%)
No data	25 (15)

HBV serology: no. pts. (%)	
Pos./neg.	46/94 (32.8/67.2)
No data	26 (15.6)

HCV serology no. pts. (%)	
Pos./neg.	4/129 (3/97)
No data	33 (19.9)

Any serological syphilis positive test: no. pts. (%)	
Pos./neg.	56/83 (40.3/59.7)
no data	27 (16.2)

**Table 2:** HPV detection in oral and anal swabs of HIV-positive homosexual men.

	Oral swab (baseline)		Oral swab (follow-up)		Anal swab (baseline)		Anal swab (follow-up)	
Positives	27	20.1%	23	21.3%	147	88.6%	101	86.3%

Genotyped	18	66.7%	19	82.6%	112	76.2%	70	69.3%

High-Risk	2	11.1%	7	36.8%	27	24.1%	11	15.7%

Low-risk	12	66.6%	5	26.3%	65	58%	49	70%

Negatives	107	79.9%	85	78.7%	19	11.4%	16	13.7%

Total evaluable specimens	134		108		166		117	

Invalid Samples*	32	19%	9	7.7%	0		0	

Rates of positive and negative specimens calculated based on total evaluable specimens. Rate of high-risk type calculated based on genotyped strains.

*absence of beta-globin in the sample

**Table 3:** Persistence of HPV in the anal area according to the risk level of the viral genotype.

Risk level in 1^st ^anal sample	Persistence			
	**no**	**yes**	**total**	**%**

LR	10	33	43	76.7%

undetermined	3	8	11	72.7%

HR	4	10	14	71.4%

Total	17	51	68	75.0%

HPV genotypes are grouped by oncogenic risk or are indicated as negative HPV samples. LR: low-risk; undetermined: undetermined-risk; possible HR: possible high-risk; HR: high-risk. Table cells contain counts, i.e., numbers of patients. The rate (percentage) of persistence is indicated by oncogenic risk. No difference was detected among the various rates (Pearson's Chi^2 ^(3) = 0.1397, P-value = 0.987).

**Figure 2:**
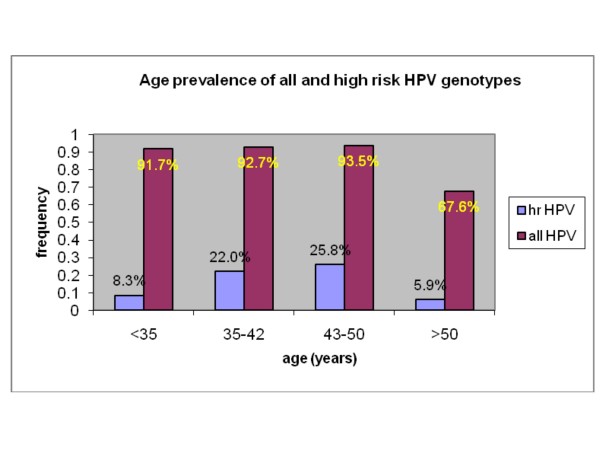
**Prevalence of all HPV genotypes in the anal area and of high-risk genotypes in four age groups**. Blue: high-risk HPV genotypes; red: all HPV genotypes. The percentages refer to the numbers of patients included in the relevant age group: 33 (< 35 years), 41 (35-42 years), 31 (43-50 years), and 34 (> 50 years). The age group delimiters are the 25th, 50th, 75th percentiles.

The prevalence of HPV anal infection and the HR genotype prevalence in various age groups are reported in Figure 2. These data suggest the existence a high anal prevalence of all HPV genotypes, and a decrease of this prevalence in the > 50 years age group. Likewise, the HR genotype prevalence is higher in the younger age groups, peaking (> 25%) in the 43-50 years age group and then declining. The changes of high-risk genotypes associated with age were not statistically significant but were close to the cut-off level [χ^2 ^(3 d.o.f.) = 7.63, p-value = 0.054].

A bivariate pairwise correlation analysis was performed to explore relationships between the main variables. A positive correlation between positive results from any syphilis diagnostic test and the presence of HR HPV genotypes in anal swabs was found, and this correlation was statistically significant (p = 0.0346). Positive syphilis tests were positively related to age (p = 0.0169) and positive HCV serology (p = 0.016). Anal swab HPV detection at either time-point was positively related to higher HIV plasma viral load (p = 0.0184) and negatively to antiretroviral therapy (p = 0.0419).

**Figure 3:**
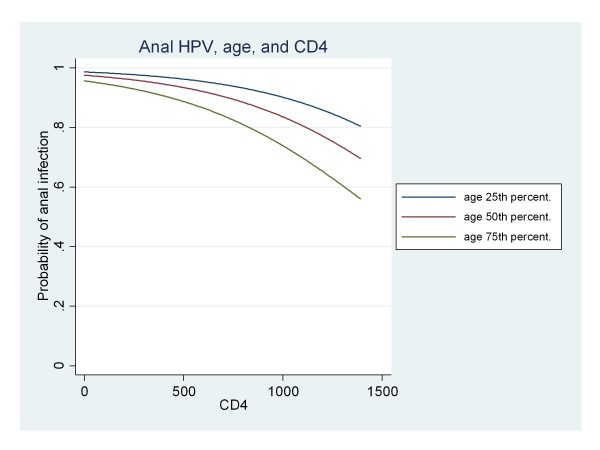
**Result of multivariate logistic analysis**. According to the final model, the probability of anal HPV infection is plotted versus the CD4 count. In order to take account of the patients' ages, three separate lines are depicted corresponding to representative age points. The 25^th ^percentile corresponds to an age of 34 years, the 50^th ^percentile (median) to 42 years, and the 75^th ^percentile to 50 years.

When the logistic multivariate analysis was performed, after stepwise elimination of the covariates with a p-value > 0.1, only two significant covariates, "age" and "CD4", were retained in the final model (Table 4, and Figure 3). The relationship between anal HPV infection and age was negative (decreasing) as was the relationship with anal HPV infection and CD4 lymphocyte count.

**Table 4:** Multivariable logistic analysis

anal_HPV	Coef.	Std. Err.	Z	p-value	95% Conf. Interval	
Age	-0.07322	0.024486	-2.99	0.003	-0.12121	-0.02523

CD4	-0.00204	0.001008	-2.02	0.043	-0.00402	-6.5E-05

Intercept	6.739866	1.527239	4.41	0.000	3.746532	9.733199

Dependent variable: anal_hpv. The number of observations used by the model was 136. Based on LR Chi^2 ^(2) = 12.62, the model was significant as quantified by a p-value = 0.0018.

HHV-8 oral shedding was positively related to HPV infection in anal area (χ^2 ^(1 d.o.f.) = 8.03, p-value = 0.0046). Moreover, a significant correlation was found between the persistence of HHV-8 shedding and HIV log10 (viral load) by logistic bivariate analysis (OR of HHV-8 persistence for 1 log increase of HIV viral load = 1.725 ± 0.397, p-value = 0.018).

## Discussion

The few studies that have analysed concurrent or sequential oral and anogenital HPV infections have examined similar study populations: high-risk sex workers, women with a history of cervical HPV infection, and HIV-positive women [15-18]. In the present longitudinal study conducted among HIV-infected MSM, we have found a high prevalence of HPV infection in the anal area, both at initial screening (88.6%) and follow-up (86.3%). These findings are consistent with those of two other longitudinal studies conducted in HIV MSM, suggesting that HIV infection not only increases susceptibility to persistent HPV but also increases the risk of acquisition of new HPV infections and the risk of reactivation of latent infections [9,28]. Our overall HPV anal prevalence data are consistent with those of earlier studies conducted in HIV-infected populations [7,29-31]. However, compared to previous studies, we have found a lower rate of HR types and a higher prevalence of LR or possible HR types. This observation is not surprising because the prevalence of infection with HR HPV types may differ substantially by geographic location [32]. When analysing specific HPV types, we found that the low-risk HPV-6 type was the most common HPV type (13% of specimens at screening and 16% at follow-up), followed by HPV-16 (13 and 7%), HPV-11 (12 and 3%), HPV-66 (11 and 7%) and HPV-61 (12 and 5%). A higher prevalence of HR types has been found in anal samples from HIV-infected patients in previous studies, with rates of up to 70% observed [7]. Conversely, low-risk genotypes have been found in higher proportions in heterosexual men with no other sexually transmitted diseases and in a small dataset of HIV-infected patients [8,33]. A number of subjects had anal infections with an HPV strain with an undetermined risk level (17.8% at baseline and 14.2% at follow up among genotyped strains).

In a more recent report on HIV-infected MSM [9], most patients (90.9%) were infected with multiple HPV types in the anal canal, with a median number of 5 HPV types per sample (range: 0-18). The median number of HR HPV types was 5 per sample (range: 0-12). The different methods used for HPV typing by the authors of these studies makes it difficult to compare their results with the results of the present study. These authors obtained genotypes using the reverse line-blot detection system [34] that is able to identify 36 genital HPV types; samples that were not positive for any of these types were considered HPV negative. This method enabled the authors to demonstrate the presence of co-infection. We chose a population sequencing method, which is less able to demonstrate viral co-infections, to be able to find rare genotypes. However, minority genotypes are easily overlooked. Nevertheless, in our study, multiple HPV infections, which can be identified sometimes during editing of the electropherograms as multiple sequences, appear to occur less frequently than in the HIPVIRG study.

Current data on the spread of HPV infection to various body parts implicated in sexual practices in both MSM and heterosexual individuals are limited. Oral sex behaviours have been associated with oral HPV infection and the transmission of other viral infections such as HSV [13,35]. Moreover, sexual behaviours are associated with a risk of cancer at the head and neck subsites that have previously been associated with HPV infection [36]. In the present study, the prevalence of HPV in oral samples was 20.1% at screening and 21.3% at follow-up. There are limited data in the literature concerning the relationship between oral and cervical HPV infection, an emerging area of research given the potentially increased risk of HPV-associated oropharyngeal cancers in HIV-positive individuals [37]. In a study of 30 HIV-positive women from South Africa who were naïve to ART and had a CD4 count of less than 300, oral HPV DNA was detected in 20% of oral specimens from the women and in 97% of the cervical specimens from the same women [38]. A limited correlation between oral HPV types and those identified in the cervical mucosa was found. In a larger study of ART-experienced women from the U.S.A., HIV-positive women were more likely to have detectable oral HPV infection than HIV-negative women (33 vs. 15%, p = 0.016) [39]. However, the prevalence of oral HPV infection over six months was significantly less than for cervical infection (p < 0.0001). These data suggest that while oral HPV infection is more common among HIV-positive women compared with HIV-negative women, the incidence and natural history of HPV infection at these two mucosal sites are not highly correlated. A similar relationship was noted in the past between cervical HPV infection and anal HPV infection in HIV-positive women [40]; while anal infection was more common than cervical infection, the HPV types detected at the two sites were different in the majority of the women.

There are fewer studies on HPV prevalence in oral specimens from men and these studies are difficult to compare. HPV infection was detected in 4.8% of 332 control patients from an outpatient clinic and in 2.9% of 210 college-aged men (age range: 18-23 years) [41]. Oral sex and open-mouthed kissing were associated with the development of oral HPV infection. Oral HPV infection is strongly associated with oropharyngeal cancer among subjects with or without the established risk factors of tobacco and alcohol use [42]. Data from another study show that HPV is detectable in clinically normal oral mucosa in approximately one third of the general population, while the detection rate observed in HIV-positive individuals is up to 60% [43]. We found the prevalence of HPV in oral specimens of HIV-infected MSM to be around 20% and we did not find genotype concordance between oral and anal HPV genotypes. Taking into account potential bias due to the lack of information from the 14.5% inadequate samples, this observation is higher than that seen in other studies (5.5% reported by Fakhry in 2006), and our data are similar to those reported by Palesky and colleagues in HIV-positive women [40].

Our findings show that the prevalence of HR HPV genotypes was highest in the 43-50 year old age group, where it was over 25%. The persistence of HR genotypes (71.4%) did not exceed the persistence of low-risk genotypes (76.7%), although further surveillance of this cohort will be necessary to understand of the determinants of HPV persistence. The presence of an HPV-positive anal swab was negatively related to age, CD4 count and antiretroviral treatment, and positively related to HIV viral load. The negative correlation between anal HPV infection and CD4 count, albeit expected, warrants further study and corresponds with the positive correlation with HIV plasma viral load. These observations support a role for HIV viral replication and immunodeficiency in affecting susceptibility to infection with all genotypes of HPV infection in the anal area or the ability to clear HPV.

The relationship between anal HPV infection and age was negative (decreasing) both by linear regression (using age as a dependent variable) and by logistic regression. However, we interpret this finding to be somewhat artifactual, as the age-related prevalence of all HPV genotypes shows a plateau followed by a decline in the group older than 50 years, whereas the high-risk HPV genotype infections show a peak in the 43-50 years age group, in partial agreement with Nyitray [44]. Moreover, two subpopulations were detected: a larger and younger population with a lower antiretroviral treatment rate, less control of HIV replication, lower CD4 levels, and a higher prevalence of anal HPV infection, including high-risk genotypes, and a smaller, older subpopulation with opposite features.

The correlation between HPV infection and *T. pallidum *infection was close to the significance level and became significant when the possible high-risk genotypes 53 and 66 were included in the analysis. Syphilis (active, latent or treated) was positively related to age, HCV infection, and, unexpectedly, lower HIV plasma viral load and higher CD4 count. The latter two correlations are probably explained by the characteristics of the older HIV patients, as the older patients were more often receiving ART and as a consequence, they had lower viral loads and higher CD4 cell counts. The association between syphilis and HCV infection is somewhat unexpected, since recent data suggests that permucosal HCV transmission might result from high-risk sexual and non-injecting drug use behaviours among MSM, raising the question of the importance of sexual transmission of HCV [45].

HHV-8 oral shedding was positively related to HPV infection in anal area: HPV infection rates were 63.6% in the HHV-8-positive and 30.0% in HHV-8-negative group. It has been demonstrated that the risk of HHV-8 infection among men in the United States and Western Europe is closely related to the number of male sexual partners, and in these populations sexual intercourse is the major mode of HHV-8 transmission [46,47]. A significant correlation was also found between the persistence of HHV-8 shedding and HIV viral load. We have previously demonstrated that HHV-8 viral load persists for up to 6 months in the majority of patients with concurrent HIV viral load, whereas all of the patients on ART with undetectable HIV viral load cleared HHV-8 from the plasma after a mean period of 12 months after the first positive blood specimen [48].

Some limitations of our study should be addressed. Our data lack information regarding a number of subjects and the robustness of our correlations should be confirmed with further observations. HPV genotyping was performed using a population sequencing method that does not allow us to fully characterise co-infections and to correctly trace all genotypes found during the double sampling. Nevertheless, this method allowed us to demonstrate a high prevalence of infection by undetermined-risk HPV types.

In conclusion, the negative relation between anal HPV infection and CD4 count detected by this multivariate logistic procedure, albeit expected, deserves interest and is in agreement with the positive correlation with HIV plasma viral load found using a bivariate analysis.

## Conclusions

These observations support a role for the HIV viral replication rate and immunodeficiency in affecting patient susceptibility to infection with all genotypes of HPV infection in the anal area and with HPV clearance. Moreover, two subpopulations were detected: a larger and younger subpopulation with a lower antiretroviral treatment rate, less control of HIV replication, lower CD4 counts, and higher prevalence of anal HPV infection including high-risk genotypes, and a smaller, older subpopulation with opposite features. The increasing incidence of anal cancer in the HIV-positive population make new strategies addressing HPV infection monitoring, control and prevention necessary; further studies addressing vaccine efficacy, HPV viral load and the role of undetermined risk variants are needed.

## Potential conflicts of interest

No conflicts

## Authors' contributions

SGP designed and coordinated the study, supervised laboratory experiments, collected the data, interpreted the findings and wrote the paper; MC helped design the study, interpret the data and write the paper; RS helped design the study, managed the patients and collected the samples; CB performed laboratory experiments; SA performed laboratory experiments; FDB performed HPV sequencing; SP contributed to HPV genotyping; AB managed patients and collected the samples; AS managed patients and collected the samples; CM helped design the study and interpret the data and performed statistical analysis; GP designed and coordinated the study and interpreted the data.

All authors read and approved the final manuscript.

## Pre-publication history

The pre-publication history for this paper can be accessed here:

http://www.biomedcentral.com/1471-2334/11/150/prepub

## References

[B1] Chin-Hong PV Palefsky JM Natural history and clinical management of anal human papillomavirus disease in men and women infected with human immunodeficiency virus Clin Infect Dis 2002 35 1127 34 10.1086/344057 12384848

[B2] Frisch M Glimelius B van den Brule AJ Wohlfahrt J Melijer CJ Walboomers JM Goldman S Svensson C Adami HO Melbye M Sexually transmitted infection as a cause of anal cancer N Engl J Med 1997 337 1350 8 10.1056/NEJM199711063371904 9358129

[B3] Grulich AE van Leeuwen MT Falster MO Vajdic CM Incidence of cancers in people with HIV/AIDS compared with immunosuppressed transplant recipients: a meta-analysis Lancet 2007 370 59 67 10.1016/S0140-6736(07)61050-2 17617273

[B4] Piketty C Selinger-Leneman H Grabar S Duvivier C Bonmarchand M Abramowitz L Costagliola D Mary-Krause M FhDh-ANRS CO 4 Marked increase in the incidence of invasive anal cancer among HIV-infected patients despite treatment with combination antiretroviral therapy AIDS 2008 22 1203 11 10.1097/QAD.0b013e3283023f78 18525266

[B5] Frisch M Fenger C van den Brule AJ Sorensen P Melijer CJ Walboomers JM Adami HO Melbye M Glimelius B Variants of squamous cell carcinoma of the anal canal and perianal skin and their relation to human papillomaviruses Cancer Res 1999 59 753 7 9973228

[B6] Vajdic CM van Leeuwen MT Jin F Prestage G Medley G Hillman RJ Stevens MP Botes LP Zablotska I Tabrizi SN Grulich AE Anal human papillomavirus genotype diversity and co-infection in a community-based sample of homosexual men Sex Transm Infect 2009 85 330 5 10.1136/sti.2008.034744 19342375

[B7] Palefsky JM Holly EA Ralston ML Jay N Prevalence and risk factors for human papillomavirus infection of the anal canal in human immunodeficiency virus (HIV)-positive and HIV-negative homosexual men J Infect Dis 1998 177 361 7 10.1086/514194 9466522

[B8] Pierangeli A Scagnolari C Degener AM Bucci M Ciardi A Riva E Indinnimeo M Mancini G D'Ettorre G Vullo V Antonelli G Type-specific human papillomavirus-DNA load in anal infection in HIV-positive men AIDS 2008 22 1929 35 10.1097/QAD.0b013e32830fbd7a 18784456

[B9] de Pokomandy A Rouleau D Ghattas G Vézina S Coté P Macleod J Allaire G Franco EL Coutlée F HIPVIRG Study Group Prevalence, clearance, and incidence of anal human papillomavirus infection in HIV-infected men: the HIPVIRG cohort study J Infect Dis 2009 199 965 73 10.1086/597207 19239366

[B10] Gillison ML Human papillomavirus-associated head and neck cancer is a distinct epidemiologic, clinical, and molecular entity Semin Oncol 2004 31 744 754 10.1053/j.seminoncol.2004.09.011 15599852

[B11] Schwartz SM Daling JR Doody RD Wipf GC Carter JJ Madeleine MM Mao EJ Fitzgibbons ED Huang S Beckmann AM McDougall JK Galloway DA Oral cancer risk in relation to sexual history and evidence of human papillomavirus infection J Natl Cancer Inst 1998 90 1626 1636 10.1093/jnci/90.21.1626 9811312

[B12] Coutlée F Trottier AM Ghattas G Leduc R Toma E Sanche G Rodrigues I Turmel B Allaire G Ghadirian P Risk factors for oral human papillomavirus in adults infected and not infected with human immunodeficiency virus Sex Transm Dis 1997 24 23 31 10.1097/00007435-199701000-00006 9018780

[B13] Kreimer AR Alberg AJ Daniel R Gravitt PE Viscidi R Garrett ES Shah KV Gillison ML Oral human papillomavirus infection in adults is associated with sexual behavior and HIV serostatus J Infect Dis 2004 189 686 698 10.1086/381504 14767823

[B14] Chaturvedi AK Engels EA Anderson WF Gillison ML Incidence trends for human papillomavirus-related and -unrelated oral squamous cell carcinomas in the United States J Clin Oncol 2008 26 612 619 10.1200/JCO.2007.14.1713 18235120

[B15] Badaracco G Venuti A Di Lonardo A Scambia G Mozzetti S Benedetti Panici P Mancuso S Marcante ML Concurrent HPV infection in oral and genital mucosa J Oral Pathol Med 1998 27 130 134 9563805 10.1111/j.1600-0714.1998.tb01928.x

[B16] Cañadas MP Bosch FX Junquera ML Ejarque M Font R Ordonez E de Sanjose S Concordance of prevalence of human papillomavirus DNA in anogenital and oral infections in a high-risk population J Clin Microbiol 2004 42 1330 1332 10.1128/JCM.42.3.1330-1332.2004 15004111 PMC356845

[B17] Kellokoski JK Syrjanen SM Chang F Yliskoski M Syrjanen KJ Southern blot hybridization and PCR in detection of oral human papillomavirus (HPV) infections in women with genital HPV infections J Oral Pathol Med 1992 21 10 459 64 10.1111/j.1600-0714.1992.tb00975.x 1334147

[B18] Fakhry C D'souza G Sugar E Weber K Goshu E Minkoff H Wright R Seaberg E Gillison M Relationship between prevalent oral and cervical human papillomavirus infections in human immunodeficiency virus-positive and -negative women J Clin Microbiol 2006 44 12 4479 85 10.1128/JCM.01321-06 17021055 PMC1698387

[B19] Katz MH McFarland W Guillin V Fenstersheib M Shaw M Kellogg T Lemp GF MacKellar D Valleroy LA Continuing high prevalence of HIV and risk behaviors among young men who have sex with men: the young men's survey in the San Francisco Bay Area in 1992 to 1993 and in 1994 to 1995 J Acquir Immune Defic Syndr Hum Retrovirol 1998 19 178 81 10.1097/00042560-199810010-00012 9768628

[B20] Schwarcz SK Kellogg TA McFarland W Louie B Klausner J Withum DG Katz MH Characterization of sexually transmitted disease clinic patients with recent human immunodeficiency virus infection J Infect Dis 2002 186 1019 22 10.1086/342954 12232844

[B21] Bernstein Kyle T Stephens Sally C Barry Pennan M Kohn Robert Philip Susan S Liska Sally Klausner Jeffrey D *Chlamydia trachomatis *and *Neisseria gonorrhoeae *Transmission from the Oropharynx to the Urethra among Men Who Have Sex with Men Clin Infect Dis 2009 49 1793 7 10.1086/648427 19911970

[B22] Ting Y Manos MM Innis MA, Gelfond DH, Sninsky JJ, White TJ Detection and typing of genital human papillomavirus PCR protocol: a guide to methods and applications 1990 San Diego: Academic Press 356 7

[B23] Grainge MJ Seth R Guo L Neal KR Coupland C Vryenhoef P Johnson J Jenkins D Cervical Human Papillomavirus Screening among Older Women Emerging Infectious Diseases 2005 11 1680 1685 http://www.cdc.gov/eid 16318718 10.3201/eid1111.050575 PMC3367359

[B24] de Roda Husman AM Walboomers JM van den Brule AJ Meijer CJ Snijders PJ The use of general primers GP5 and GP6 elongated at their 3' ends with adjacent highly conserved sequences improves human papillomavirus detection by PCR J Gen Virol 1995 76 1057 62 10.1099/0022-1317-76-4-1057 9049358

[B25] Auvert B Lissouba P Cutler E Zarca K Puren A Taljaard D Association of oncogenic and nononcogenic Human Papillomavirus with HIV Incidence J Acquir Immune Defic Syndr 2010 53 111 116 10.1097/QAI.0b013e3181b327e7 19779357 PMC3203884

[B26] White IE Campbell TB Quantitation of Cell-Free and Cell-Associated Kaposi's Sarcoma-Associated Herpesvirus DNA by Real-Time PCR J Clin Microb 2000 38 5 1992 95 10.1128/jcm.38.5.1992-1995.2000 PMC86648 10790138

[B27] Poole LJ Zong DJ Ciufo DM Alcendor DJ Cannon JS Ambinder R Orenstein JM Reitz MS Hayward GS Comparison of genetic variability at multiple loci across the genomes of the major subtypes of Kaposi's Sarcoma-Associated Herpesvirus reveals evidence for recombination and for two distinct types of open reading frame K15 alleles at the right-hand end J Virol 1999 73 6646 60 10400762 10.1128/jvi.73.8.6646-6660.1999 PMC112749

[B28] Critchlow CW Hawes SE Kuypers JM Goldbaum GM Holmes KK Surawicz CM Kiviat NB Effect of HIV infection on the natural history of anal human papillomavirus infection AIDS 1998 12 1177 84 10.1097/00002030-199810000-00010 9677167

[B29] Van der Snoek EM Niesters HG Mulder PG van Doornum GJ Osterhaus AD van der Meijden WI Human papillomavirus infection in men who have sex with men participating in a Dutch gay-cohort study Sex Transm Dis 2003 30 639 644 10.1097/01.OLQ.0000079520.04451.59 12897686

[B30] Orlando G Tanzi E Beretta R Amendola A Fasolo MM Bianchi S Cellerino P Mazza F Zappa A Rizzardini G Human papillomavirus genotypes and anal related lesions among HIV-1-infected men in Milan, Italy J Acquir Immune Defic Syndr 2008 47 129 131 10.1097/QAI.0b013e318156ec7b 18156995

[B31] Kiviat N Critchlow C Holmes K Kuipers KK Sayer J Dunphy C Surawicz C Kirby P Wood R Daling JR Association of anal dysplasia and human papillomavirus with immunosuppression and HIV infection among homosexual men AIDS 1993 7 43 9 10.1097/00002030-199301000-00007 8382927

[B32] Nyitray AG Smith D Villa L Lazcano-Ponce E Abrahamsen M Papenfuss M Giuliano AR Prevalence of and risk factors for anal human papillomavirus infection in men who have sex with women: a cross-national study J Infect Dis 2010 201 10 1498 508 10.1086/652187 20367457 PMC2856726

[B33] Nielson CM Flores R Harris RB Abrahamsen M Papenfuss MR Dunne EF Markowitz LE Giuliano AR Human papillomavirus prevalence and type distribution in male anogenital sites and semen Cancer Epidemiol Biomarkers Prev 2007 16 1107 1114 10.1158/1055-9965.EPI-06-0997 17548671

[B34] Gohy L Gorska I Rouleau D Ghattas G Pokomandy A Vézina S Coté P Macleod J Allaire G Hadjeres R Kornegay JR Franco E HIPVIRG Study Group Coutlée F Genotyping of human papillomavirus DNA in anal biopsies and anal swabs collected from HIVseropositive men with anal dysplasia J Acquir Immune Defic Syndr 2008 49 32-9 21 10.1097/QAI.0b013e318183a905 18667921

[B35] Edwards S Carne C Oral sex and the transmission of viral STIs Sex Transm Infect 1998 74 6 10 10.1136/sti.74.1.6 9634307 PMC1758078

[B36] Heck JE Berthiller J Vaccarella S Winn DM Smith EM Shan'gina O Schwartz SM Purdue MP Pilarska A Eluf-Neto J Menezes A McClean MD Matos E Koifman S Kelsey KT Herrero R Hayes RB Franceschi S Wünsch-Filho V Fernández L Daudt AW Curado MP Chen C Castellsagué X Ferro G Brennan P Boffetta P Hashibe M Sexual behaviours and the risk of head and neck cancers: a pooled analysis in the International Head and Neck Cancer Epidemiology (INHANCE) consortium Int J Epidemiol 2010 39 1 166 81 10.1093/ije/dyp350 20022926 PMC2817092

[B37] Palesky J Human papillomavirus-related disease in people with HIV Curr Opin HIV AIDS 2009 4 1 52 6 10.1097/COH.0b013e32831a7246 19339939 PMC2756707

[B38] Richter KL van Rensburg EJ van Heerden WF Boy SC Human papilloma virus types in the oral and cervical mucosa of HIV-positive South African women prior to antiretroviral therapy J Oral Pathol Med 2008 37 9 555 9 10.1111/j.1600-0714.2008.00670.x 18355174

[B39] D'Souza G Fakhry C Sugar EA Seaberg EC Weber K Minkoff HL Anastos K Palefsky JM Gillison ML Six-month natural history of oral versus cervical human papillomavirus infection Int J Cancer 2007 121 143 50 10.1002/ijc.22667 17354235

[B40] Palefsky JM Holly EA Ralston ML Da Costa M Greenblatt RM Prevalence and risk factors for anal human papillomavirus infection in human immunodeficiency virus (HIV)-positive and high-risk HIV-negative women J Infect Dis 2001 183 3 383 91 10.1086/318071 11133369

[B41] D'Souza G Agrawal Y Halpern J Bodison S Gillison ML Oral sexual behaviors associated with prevalent oral human papillomavirus infection J Infect Dis 2009 199 9 1263 9 10.1086/597755 19320589 PMC4703086

[B42] D'Souza G Kreimer AR Viscidi R Pawlita M Fakhry C Koch WM Westra WH Gillison ML Case-control study of human papillomavirus and oropharyngeal cancer N Engl J Med 2007 356 19 1944 56 10.1056/NEJMoa065497 17494927

[B43] Adler-Storthz K Ficarra G Woods KV Gaglioti D Di Pietro M Shillitoe EJ Prevalence of Epstein-Barr virus and human papillomavirus in oral mucosa of HIV-infected patients J Oral Pathol Med 1992 21 164 170 10.1111/j.1600-0714.1992.tb00095.x 1318379

[B44] Nyitray AG Carvalho da Silva RJ Baggio ML Lu B Smith D Abrahamsen M Papenfuss M Villa LL Lazcano-Ponce E Giuliano AR Age-Specific Prevalence of and Risk Factors for Anal Human Papillomavirus (HPV) among Men Who Have Sex with Women and Men Who Have Sex with Men: The HPV in Men (HIM) Study J Infect Dis 2011 203 49 57 10.1093/infdis/jiq021 21148496 PMC3086435

[B45] van de Laar TJ Matthews GV Prins M Danta M Acute hepatitis C in HIV-infected men who have sex with men: an emerging sexually transmitted infection AIDS 2010 24 12 1799 812 10.1097/QAD.0b013e32833c11a5 20601854

[B46] Kedes DH Operskalski E Busch M Kohn R Flood J Ganem D The seroepidemiology of human herpesvirus 8 (Kaposi's sarcoma-associated herpesvirus):Distribution of infection in KS risk groups and evidence for sexual transmission Nat Med 1996 2 918 24 10.1038/nm0896-918 8705863

[B47] Martin JN Ganem DE Osmond DH Page-Shafer KA Macrae D Kedes DH Sexual transmission and the natural history of human herpesvirus 8 infection N Engl J Med 1998 338 948 54 10.1056/NEJM199804023381403 9521982

[B48] Parisi SG Boldrin C Andreis S Ferretto R Fuser R Malena M Manfrin V Panese S Scaggiante R Dori L Sarmati L Biasolo MA Nicastri E Andreoni M Cruciani M Palù G KSHV DNA viraemia correlates with low CD4+ cell count in Italian males at the time of HIV infection diagnosis J Med Virol 2011 83 384 390 10.1002/jmv.21987 21264857

